# Understanding of Clinical Trials Among Patients With Cancer and Their Relatives

**DOI:** 10.1001/jamanetworkopen.2024.57020

**Published:** 2025-01-28

**Authors:** Pınar Kubilay Tolunay, Cihan Erol, Seda Kahraman, Seher Yıldız Tacar, Erkan Özcan, Fatma Buğdaycı Başal, Fatih Köse, Mehmet Ali Nahit Şendur, Deniz Tural, İrfan Çiçin, Berna Öksüzoğlu, Saadettin Kılıçkap, Yüksel Ürün

**Affiliations:** 1 Ankara University Faculty of Medicine, Department of Medical Oncology, Ankara, Turkey; 2 Ankara Yıldırım Beyazıt University Faculty of Medicine Department of Medical Oncology, Ankara, Turkey; 3 Bakırköy Dr Sadi Konuk Training and Research Hospital Department of Medical Oncology Istanbul, Turkey; 4 Trakya University School of Medicine, Department of Medical Oncology, Edirne, Turkey; 5 Dr Abdurrahman Yurtaslan Ankara Oncology Training and Research Hospital, Department of Medical Oncology, Ankara, Turkey; 6 Baskent University, Dr Turgut Noyan Training and Research Center, Adana, Turkey; 7 İstinye University Faculty of Medicine, Department of Medical Oncology, Istanbul, Turkey

## Abstract

**Importance:**

Clinical trials are vital for advancing cancer treatments and improving patient outcomes. Understanding the factors that influence participants’ decision-making is critical for enhancing trial recruitment.

**Objective:**

To evaluate the attitudes of patients with cancer and their relatives toward clinical trial participation, identifying key barriers and motivators that affect their willingness to engage in such trials.

**Design, Setting, and Participants:**

This cross-sectional survey study was conducted between April 2020 and April 2021. Face-to-face questionnaires were administered by physicians across 6 tertiary hospital medical oncology departments in Turkey. Adults with cancer and their relatives were recruited. Data were analyzed from April to December 2021.

**Exposure:**

Participants’ knowledge, perceptions, and motivations regarding clinical trial participation were assessed through a structured questionnaire.

**Main Outcomes and Measures:**

Participants’ demographic information, their willingness to participate in clinical trials, their perceptions about the clinical trial participation, and the facilitators and barriers to participation.

**Results:**

A total of 978 participants were surveyed, with a median (range) age of 52 (18-82) years; 485 (49.6%) were male and 479 (49.0%) female. Of these, 578 (59.1%) were patients with cancer and 382 (39.1%) family members. Prior clinical trial experience was reported by 174 participants (17.8%), and 428 (43.8%) expressed a willingness to participate in clinical trials. Participants well-informed about clinical trials showed higher willingness (50 of 87 [57.5%] very willing) compared with those with no knowledge (27 of 303 [8.9%] very willing) (χ^2^ = 275.095; *P* < .001). Greater willingness was observed in participants from less developed cities compared with the most developed cities (88 of 321 [27.4%] vs 94 of 615 [15.3%]; χ^2^ = 21.093; *P* < .001), in individuals with a high school degree or greater compared with those with less than a high school degree (105 of 489 [21.5%] vs 76 of 452 [16.8%]; χ^2^ = 33.311; *P* < .001), in those with monthly incomes above compared with below the poverty line (81 of 409 [19.8%] vs 100 of 512 [19.5%]; χ^2^ = 16.145; *P* = .003), in those without prior cancer treatment compared with those with prior cancer treatment (125 of 591 [21.2%] vs 40 of 289 [13.8%]; χ^2^ = 13.801; *P* = .008), and in participants with prior trial experience compared with those without (74 of 166 [44.6%] vs 111 of 786 [14.1%]; χ^2^ = 87.771; *P* < .001). Participants were motivated by potential personal health benefits (604 [61.8%]) and access to new treatments (522 [53.4%]). The primary concerns included potential adverse effects (555 [56.7%]), feeling like a “test subject” (284 [29.0%]), and the risk of receiving a placebo (197 [20.1%]).

**Conclusions and Relevance:**

In this survey study of patients with cancer and their relatives, significant gaps in knowledge and persistent concerns about clinical trial safety were highlighted, impacting participation. Addressing these concerns through targeted education and transparent communication is essential for improving participation rates and ensuring more inclusive cancer research.

## Key Points

**Question:**

What are the attitudes toward and perceptions of participating in clinical trials among patients with cancer and their relatives, and what factors are associated with their willingness or hesitation to engage?

**Findings:**

In this survey study of 978 patients with cancer and their relatives, willingness to participate and knowledge about clinical trials were limited. Significant gaps in understanding and persistent concerns about participation remain.

**Meaning:**

These findings underscore the importance of patient education, transparent communication, and trust-building by health care professionals to address barriers and enhance participation in clinical trials, ultimately advancing cancer research and improving patient outcomes.

## Teaser

This survey study evaluates the knowledge, perceptions, and motivations among patients with cancer and their relatives in Turkey regarding participation in clinical trials of cancer treatments.

## Introduction

Clinical trials are indispensable for the development of novel treatments and the enhancement of cancer care outcomes, forming the bedrock of advancements in oncology research.^1^ Despite their pivotal role in medical science, the recruitment and engagement of patients in these trials present substantial challenges. Understanding the intricate interplay of barriers and motivations that influence patients’ decisions to enroll is crucial for enhancing inclusivity and ensuring the success of oncology research.^2,3^

While participation in clinical trials can offer patients access to state-of-the-art treatments and the potential for advancements in their care, it also involves inherent risks, such as potential adverse effects and uncertainties regarding treatment efficacy.^4,5,6^ Although it is believed that participants in clinical research receive more effective treatments, studies have shown that state-of-the-art treatments in clinical trials may not provide a survival advantage over standard therapies; nevertheless, patients are able to make an altruistic contribution to advancing knowledge and potentially benefiting future patients.^4,5,7^ The decision to participate is highly individualized, influenced by a multitude of factors including financial constraints, emotional and moral considerations, and historical mistrust due to past injustices and inequalities in medical research. Concerns about unknown adverse effects and logistical difficulties further complicate the decision-making process, often deterring participation.^8,9,10,11^

This study aimed to assess the attitudes of patients with cancer and their relatives toward clinical trial participation. By identifying the barriers and motivating factors specific to this population, we seek to inform strategies that could enhance participation rates. A deeper understanding of these influences will contribute to more inclusive and effective clinical trials, ultimately improving patient outcomes and advancing cancer treatment.

## Methods

We conducted a cross-sectional survey to assess the attitudes and perceptions of patients with cancer and their family caregivers regarding participation in clinical trials. The study specifically targeted individuals diagnosed with cancer, as well as their family members, to gain a comprehensive understanding of attitudes toward clinical trial participation. It was conducted across 6 tertiary hospitals with oncology clinics and clinical trial centers in Turkey, including Ankara University Faculty of Medicine, Ankara Bilkent City Hospital, Istanbul Bakirkoy Dr Sadi Konuk Training and Research Hospital, Trakya University Faculty of Medicine, Dr Abdurrahman Yurtaslan Ankara Oncology Training and Research Hospital, and Adana Baskent University Hospital. The survey was conducted from April 1, 2020, to April 31, 2021. Eligible participants included individuals diagnosed with cancer or those with a family member diagnosed with cancer, who were fluent in Turkish, and were aged at least 18 years. Participants included both newly diagnosed and previously treated patients as well as those who had participated in clinical research. During cancer patients’ outpatient visits, physicians invited participants to take part in the survey. Participants were informed that their participation was completely voluntary and that they had the right to withdraw from the survey at any time without any consequences. After beginning the survey, 24 participants withdrew and were therefore not included in the results. Written informed consent was obtained from individuals who consented to participate. The questionnaire was administered face-to-face by medical oncologists (P.K.T., C.E., S.K., S.Y.T., E.Ö., F.B.B., and F.K.) in outpatient medical oncology clinics. No stipend was provided to the participants for their involvement. The study received ethical approval from the Ankara University Faculty of Medicine Human Research Ethics Committee and approval from the institutional authorities at participating sites. The principles outlined in the Declaration of Helsinki were followed. The study was conducted in accordance with the ethical guidelines and best practices outlined in the American Association for Public Opinion Research (AAPOR) reporting guideline.^12^

The questionnaire was meticulously designed, drawing from established methods in prior studies, and concentrated on gathering demographic data alongside assessing participants’ willingness to engage in clinical trials.^13,14,15,16,17^ A pilot survey was conducted in Turkish with 30 participants to ensure that the survey items were clear and easily understood. This study was reported in accordance with the Strengthening the Reporting of Observational Studies in Epidemiology (STROBE) guidelines for cross-sectional studies.^18^

### Statistical Analysis

Data were analyzed using SPSS version 25.0 (IBM Corp). Categorical data were expressed as frequency and percentage. Comparisons between groups were carried out using χ^2^ test for categorical variables. A 2-tailed *P* < .05 was accepted as the level of significance.

## Results

### Participant Characteristics

A total of 978 individuals participated in this study, ranging in age from 18 to 82 years, with a median age of 52 years. The sample consisted of 485 (49.6%) male and 479 (49.0%) female participants. Among participants, 174 (17.8%) had previous experience with clinical trials, providing valuable insights into factors influencing repeat participation, which could guide future recruitment strategies. The respondents included 578 (59.1%) patients with cancer and 382 (39.1%) family members. Participants were categorized based on the socioeconomic development levels of their provinces, as defined by the State Planning Organization of Turkey. Low income was defined as having an income below the poverty line according to the Turkish Statistical Institute. Participants’ characteristics are summarized in Table 1.

**Table 1.** Respondent Characteristic

Characteristic	Respondents, No. (%)
Gender	
Male	485 (49.6)
Female	479 (49)
Missing	14 (1.4)
Age, y	
Median (range)	52 (18-82)
≥65	210 (21.5)
<65	751 (78.8)
Missing	17 (1.7)
Residence	
Most developed cities	632 (64.6)
Less developed cities	328 (33.5)
Missing	18 (1.8)
Residence type	
City centers	671 (68.6)
Town or village	289 (29.6)
Missing	18 (1.8)
Education	
Less than high school	465 (47.5)
High school and above	500 (51.1)
Missing	13 (1.3)
Marital status	
Married	763 (78)
Single	212 (21.7)
Missing	3 (0.3)
Employment status	
Employed	281 (28.7)
Unemployed or retired	689 (70.4)
Missing	8 (0.8)
Prior clinical trial participation	
Yes	174 (17.8)
No	802 (82.0)
Missing	2 (0.2)
Cancer patient or family member	
Patient	578 (59.1)
Family member	382 (39.1)
Missing	18 (1.8)
Financial status	
Monthly income below poverty line	532 (54.4)
Monthly income above poverty line	411 (42.0)
Missing	35 (3.6)
Household members	
≤3	522 (53.4)
>3	374 (38.2)
Missing	82 (8.4)
First time visitors	
Yes	336 (34.4)
No	634 (64.8)
Missing	8 (0.8)
Cancer type	
Gastrointestinal	306 (31.3)
Breast	241 (24.6)
Lung	173 (17.7)
Genitourinary	167 (17.1)
Head and neck	22 (2.2)
Lymphoma	18 (1.8)
Other malignant neoplasms	33 (3.5)
Missing	18 (1.8)
Cancer stage	
Early stage or remission	311 (31.8)
Advanced stage	296 (30.3)
Not known	273 (27.9)
Missing	98 (10.0)
History of cancer treatment	
Yes	300 (30.7)
No	602 (61.6)
Missing	76 (7.8)
Actively receiving cancer treatment	
Yes	642 (65.6)
No	277 (28.3)
Missing	59 (6.0)
Self-rated health status	
Good or excellent	507 (51.8)
Fair or poor	450 (46.0)
Missing	21 (2.1)
Family member or friend had cancer treatment before	
Yes	509 (52.0)
No	370 (37.8)
Not known	71 (7.3)
Missing	28 (2.9)
Asked to participate in clinical trial	
Yes	262 (26.8)
No	585 (59.8)
Not sure	108 (11.0)
Missing	23 (2.4)
Willingness to participate in clinical trials	
Likely to participate	428 (43.8)
Neither likely nor unlikely	297 (30.4)
Unlikely to participate	229 (23.4)
Missing	24 (2.5)
Knowledge about clinical trials	
Well-informed	92 (9.4)
Somewhat informed	299 (30.6)
Not informed (no or very little knowledge)	578 (59.0)
Missing	10 (1.0)

### Willingness to Participate in Clinical Trials

Among those surveyed, 229 (23.4%) were unwilling to participate in a clinical trial if given the opportunity, 297 (30.4%) were undecided, and 428 (43.8%) were willing to participate. Of the 428 respondents willing to participate, 252 (58.9%) were the patients themselves, 164 (38.3%) were relatives, 112 (26.2%) had prior clinical research experience, either personally or through their relative, and 293 (68.5%) were either undergoing active cancer treatment themselves or had relative who was.

Willingness to participate was significantly associated with several factors (Table 2). A well-informed understanding of clinical trials was significantly associated with greater willingness to participate, with 50 of 87 well-informed participants (57.5%) being very willing compared with only 27 of 203 those with no knowledge (8.9%) (χ^2^ = 275.095; *P* < .001). Participants living in less developed cities showed greater willingness (88 of 321 [27.4%] very willing) than those from more developed cities (94 of 615 [15.3%] very willing) (χ^2^ = 21.093; *P* < .001). Those with at least a high school education were more willing (105 of 489 [21.5%] very willing) than those with less education (76 of 452 [16.8%] very willing) (χ^2^ = 33.311; *P* < .001). Participants with household incomes above the poverty line showed slightly greater willingness (81 of 409 [19.8%] very willing) compared with those below it (100 of 512 [19.5%] very willing) (χ^2^ = 16.145; *P* = .003). Additionally, those who had not previously received cancer treatment had higher willingness (125 of 591 [21.2%] very willing) compared with those with prior treatment (40 of 289 [13.8%] very willing) (χ^2^ = 13.801, *P* = .008), and those with a friend or relative who received cancer treatment were more willing (107 of 497 [21.5%] very willing) than those without such experience (164 of 360 [17.8%] very willing) (χ^2^ = 16.984; *P* = .030). Prior experience with clinical trials was also associated with willingness to participate, with significantly higher willingness among prior trial participants (74 of 166 [44.6%] very willing) compared with those without experience (111 of 786 [14.1%] very willing) (χ^2^ = 87.771; *P* < .001).

**Table 2.** Factors Associated With Willingness to Participate in Clinical Trials

Factor	If given the opportunity, how willing would you be to participate in a clinical trial?						χ^2^	*P* value
	Total No.	Not at all willing	Not very willing	Unsure	Somewhat willing	Very willing		
How would you describe your health status?								
Very poor or poor or fair	440	35 (8.0)	72 (16.4)	156 (35.5)	106 (24.1)	71 (16.1)	11.023	.03
Good or excellent	493	47 (9.5)	70 (14.2)	135 (27.4)	133 (27.0)	108 (21.9)		
Do you have knowledge of clinical trials?								
No knowledge	303	28 (9.2)	36 (11.9)	175 (57.8)	37 (12.2)	27 (8.9)	275.095	<.001^a^
Little knowledge	264	34 (12.9)	57 (21.6)	68 (25.8)	80 (30.3)	25 (9.5)		
Some knowledge	295	19 (6.4)	44 (14.9)	43 (14.6)	107 (36.3)	82 (27.8)		
Well-informed	87	5 (5.7)	6 (6.9)	9 (10.3)	17 (19.5)	50 (57.5)		
Age group, y								
18-49	408	33 (8.1)	61 (15.0)	130 (31.9)	111 (27.2)	73 (17.9)	1.896	.76
≥50	530	52 (9.8)	78 (14.7)	162 (30.6)	132 (24.9)	106 (20.0)		
Gender								
Female	468	47 (10.0)	67 (14.3)	162 (34.6)	113 (24.1)	79 (16.9)	7.887	.10
Male	472	39 (8.3)	73 (15.5)	131 (27.8)	128 (27.1)	101 (21.4)		
Residence								
Most developed cities	615	64 (10.4)	94 (15.3)	201 (32.7)	162 (26.3)	94 (15.3)	21.093	<.001^a^
Less developed cities	321	22 (6.9)	45 (14.0)	91 (28.3)	75 (23.4)	88 (27.4)		
Education level								
Less than high school	452	49 (10.8)	61 (13.5)	176 (38.9)	90 (19.9)	76 (16.8)	33.311	<.001^a^
High school and above	489	37 (7.6)	81 (16.6)	118 (24.1)	148 (30.3)	105 (21.5)		
Monthly household income								
Below poverty line	512	56 (10.9)	69 (13.5)	173 (33.8)	114 (22.3)	100 (19.5)	16.145	.003^a^
Above poverty line	409	27 (6.6)	71 (17.4)	108 (26.4)	122 (29.8)	81 (19.8)		
First time visitors to oncology clinic								
No	616	60 (9.7)	94 (15.3)	194 (31.5)	149 (24.2)	119 (19.3)	2.318	.68
Yes	331	25 (7.6)	49 (14.8)	102 (30.8)	92 (27.8)	63 (19.0)		
Stage of illness								
Do not know	269	33 (12.3)	45 (16.7)	92 (34.2)	60 (22.3)	39 (14.5)	14.136	.08
Early stage or remission	304	28 (9.2)	40 (13.2)	91 (29.9)	90 (29.6)	55 (18.1)		
Advanced stage	290	20 (6.9)	46 (15.9)	86 (29.7)	74 (25.5)	64 (22.1)		
Have you received cancer treatment before?								
No	591	51 (8.6)	84 (14.2)	196 (33.2)	135 (22.8)	125 (21.2)	13.801	.008^a^
Yes	289	27 (9.3)	54 (18.7)	81 (28.0)	87 (30.1)	40 (13.8)		
Are you currently undergoing cancer treatment?								
No	270	28 (10.4)	51 (18.9)	86 (31.9)	68 (25.2)	37 (13.7)	8.913	.06
Yes	627	52 (8.3)	87 (13.9)	195 (31.1)	164 (26.2)	129 (20.6)		
Has any relative or friend of yours ever received cancer treatment?								
Do not know	69	6 (8.7)	8 (11.6)	34 (49.3)	14 (20.3)	7 (10.1)	16.984	.03
Yes	497	47 (9.5)	69 (13.9)	146 (29.4)	128 (25.8)	107 (21.5)		
No	360	29 (8.1)	65 (18.1)	108 (30.0)	94 (26.1)	64 (17.8)		
Have you participated in a clinical trial?								
No	786	77 (9.8)	135 (17.2)	259 (33.0)	204 (26.0)	111 (14.1)	87.771	<.001^a^
Yes	166	9 (5.4)	8 (4.8)	37 (22.3)	38 (22.9)	74 (44.6)		

^a^ Statistically significant.

### General Perceptions of Clinical Trials

When asked about their general perception of clinical trials, 361 (37.8%) said they were neutral, 268 (28.1%) had a negative general perception, and 325 (34.1%) had a positive general perception. General perception of clinical trials was associated with several factors (Table 3). Participants who disagreed with the statement “They are treated as if they are not human, but rather as guinea pigs or test subjects” were more likely to have a positive perception of clinical trials (196 of 437 [44.9%]) compared with those who were neutral (56 of 292 [19.2%]) or agreed with the statement (46 of 148 [31.1%]) (χ^2^ = 52.815; *P* < .001). Those who disagreed with “The primary purpose of developing new treatment methods/drugs is for companies to make money” showed a more positive perception (181 of 446 [40.6%]) compared with those who were neutral (88 of 343 [25.7%]) or agreed (50 of 143 [35.0%]) (χ^2^ = 21.985, *P* < .001). Those who agreed with “The primary purpose of developing new treatment methods/drugs is for doctors to make money” had the highest positive general perception (22 of 53 [41.5%]), followed by those who disagreed (259 of 680 [38.1%]) and those who were neutral (37 of 197 [18.8%]) (χ^2^ = 30.646; *P* < .001). However, the agreed group had a smaller sample size.

**Table 3.** Factors Associated With General Perception of Clinical Trials

Opinion^a^	Total No.	What is your general perception of clinical trials?			χ^2^	*P* value
		Negative	Neutral	Positive		
**Treated rather as guinea pigs or test subjects**						
Disagree	437	102 (23.3)	139 (31.8)	196 (44.9)	52.815	<.001^b^
Neutral	292	91 (31.2)	145 (49.7)	56 (19.2)		
Agree	148	41 (27.7)	61 (41.2)	46 (31.1)		
**The primary purpose of developing new treatment methods/drugs is for pharmaceutical companies to make money**						
Disagree	446	115 (25.8)	150 (33.6)	181 (40.6)	21.985	<.001^b^
Neutral	343	98 (28.6)	157 (45.8)	88 (25.7)		
Agree	143	44 (30.8)	49 (34.3)	50 (35.0)		
**The primary purpose of developing new treatment methods/drugs is for doctors to make money**						
Disagree	680	183 (26.9)	238 (35.0)	259 (38.1)	30.646	<.001^b^
Neutral	197	57 (28.9)	103 (52.3)	37 (18.8)		
Agree	53	15 (28.3)	16 (30.2)	22 (41.5)		

^a^ The statements listed in this table are direct quotations from the survey, translated from Turkish.

^b^ Statistically significant.

### Awareness and Knowledge of Clinical Trials

When participants were asked about their knowledge of clinical trials, 306 (31.2%) reported having no knowledge, 272 (27.8%) indicated they had very little knowledge, 299 (30.6%) had some knowledge, and 92 (9.4%) felt well-informed. Regarding offers to participate in clinical trials, 108 (11.0%) were unsure, 262 (26.8%) had been offered, and 585 (59.8%) had not.

When asked about the appropriateness of conducting clinical trials under government supervision and within the framework of Ministry of Health regulations, 284 (29.0%) were unsure, 657 (67.2%) agreed it was correct, and 24 (2.5%) disagreed. Additionally, 203 (20.8%) believed clinical trials involve only new drugs that have not been tested on humans before, while 566 (57.9%) did not know, and 194 (19.8%) thought this was not true. Regarding the safety of clinical trials, 117 (12.0%) considered them unsafe, 445 (45.5%) were uncertain, and 397 (40.6%) believed they were safe.

### Motivations and Concerns

Overall, 604 (61.8%) believed participating in clinical trials could improve their illness, 522 (53.4%) were motivated by access to new treatments, and 291 (29.8%) hoped to discover genetic diseases in their family. Additionally, 255 (26.1%) appreciated receiving free medications, while 246 (25.2%) valued more attentive care from health care professionals. Notably, 125 (12.8%) felt participation would not benefit them personally.

Regarding societal benefits, 603 (61.7%) believed their participation could advance science, and 540 (55.2%) thought it would benefit future patients. A large majority (888 [90.8%]) believed clinical trials benefit society.

Participants identified several risks associated with clinical trials. The most concerning were potential adverse effects (555 [56.7%]) and the chance of receiving a placebo (197 [20.1%]). Privacy concerns (111 [11.3%]), feeling like a “test subject” (284 [29.0%]), and the potential for exploitation (157 [16.1%]) were also noted. While 93 participants (9.5%) saw no risk in participating, most (885 [90.5%]) perceived some form of risk. The top 5 motivations for and concerns about clinical trial participation are shown in the Box.

Box. **Top 5 Motivations and Concerns for Clinical Trial Participation**

**Motivations**

- Improvement of illness (604 participants [61.8%])
- Access to new and effective treatments (522 participants [53.4%])
- Discovery of genetic diseases in the family (291 [29.8%])
- Receiving medications free (255 [26.1%])
- Receiving more attentive care and spending more time with health care professionals (246 [25.2%])

**Concerns**

- Adverse effects of research treatments (555 [56.7%])
- Being treated as a “test subject” (284 [29.0%])
- Chance of receiving a placebo or ineffective drug (197 [20.1%])
- Potential for exploitation (157 [16.1%])
- Breach of medical information privacy (111 [11.3%])

Participants were also asked about their opinions on clinical trial participants and the factors influencing their decision to participate in clinical trials using a Likert scale. These results are presented in Table 4.

**Table 4.** Influences and Barriers to Participating in Clinical Trials

Factor^a^	No. (%)			
	Strongly agree or agree	Undecided	Strongly disagree or disagree	Missing data
**Clinical trial participants**				
Access the best doctors	448 (45.8)	332 (33.9)	155 (15.8)	43 (4.4)
Access the best and latest treatments not otherwise available	370 (37.8)	373 (38.1)	165 (16.9)	70 (7.2)
Treated as if they were not human, but rather as guinea pigs or test subjects	152 (15.5)	299 (30.6)	447 (45.7)	80 (8.2)
Gain more information about their health condition.	629 (64.3)	210 (21.5)	74 (7.6)	65 (6.6)
Make a significant contribution to science	757 (77.4)	136 (13.9)	23 (2.4)	62 (6.3)
Risk their health	209 (21.4)	363 (37.1)	324 (33.1)	82 (8.4)
Receive more attention from doctors and other staff, and more time is dedicated to them	420 (42.9)	306 (31.3)	183 (18.7)	69 (7.1)
**Has an impact on my decision to take part in clinical research**				
Advanced stage of illness	605 (61.9)	184 (18.8)	118 (12.1)	71 (7.3)
Recommendation from a doctor I trust	743 (76.0)	99 (10.1)	71 (7.3)	65 (6.6)
Trustworthiness of the health care institution	740 (75.7)	109 (11.1)	66 (6.7)	63 (6.4)
Belief in the treatment’s potential to cure	651 (66.6)	171 (17.5)	84 (8.6)	72 (7.4)
Knowing active medication is administered instead of a placebo	494 (50.5)	308 (31.5)	75 (7.7)	101 (10.3)
Free treatment	576 (58.9)	138 (14.1)	180 (18.4)	84 (8.6)
The belief that the treatment will be effective for my disease	749 (76.6)	107 (10.9)	53 (5.4)	69 (7.1)
Having no other treatment options	642 (65.6)	182 (18.6)	70 (7.2)	84 (8.6)
Side effects related to treatment	572 (58.5)	224 (22.9)	95 (9.7)	87 (8.9)
Belief that the treatment could benefit others in the future	670 (68.5)	165 (16.9)	65 (6.6)	78 (8.0)
Positive experiences of others who participated in clinical trials	652 (66.7)	164 (16.8)	83 (8.5)	79 (8.1)
Opinions of family or close friends	572 (58.5)	168 (17.2)	161 (16.5)	77 (7.9)
Social media or internet forums	286 (29.2)	264 (27.0)	344 (35.2)	84 (8.6)
Distance of the research center from home	478 (48.9)	151 (15.4)	264 (27.0)	85 (8.7)
Duration of treatment and follow-up	580 (59.3)	149 (15.2)	168 (17.2)	81 (8.3)
Opinions/experiences of patients previously involved	663 (67.8)	140 (14.3)	98 (10.0)	77 (7.9)
Knowing the study is approved by health authorities and ethics boards	693 (70.9)	140 (14.3)	71 (7.3)	74 (7.6)

^a^ The statements listed in this table are direct quotations from the survey, translated from Turkish.

### Preferred Sources of Information for Potential Clinical Trial Participation

A significant majority (824 [84.3%]), would seek information from their attending oncologist, 217 (22.2%) would prefer information from their family physician, 464 (47.4%) would prefer information from clinical trial doctors, and 126 (12.9%) prefer information from other health care professionals. Nearly one-third (315 [32.2%]) would prefer information from patients who have participated in clinical trials. Only a minority of participants stated that they would prefer to receive information about clinical trials from disease forums and social media (34 [3.5%]), traditional media outlets (30 [3.1%]), or the internet (78 [8.0%]).

### Other Aspects of Clinical Trials Participants Considered

A total of 699 participants (71.5%) would be more inclined to participate a clinical trial if their oncologist was also the one conducting the trial. Nearly one-quarter (234 [23.9%]) said it would not influence their decision.

Regarding their interest in knowing the results after the completion of the clinical trial and once all patient outcomes are available, 782 participants (80.0%) expressed a desire to know the results. When asked if they would worry about receiving suboptimal treatment from their physician if they declined to participate in a clinical trial, 116 (11.9%) were concerned about potentially receiving inferior treatment, 235 (24.0%) were undecided, and 602 (61.6%) did not share the concern.

Overall, 143 participants (14.6%) agreed that the primary motive for development new treatments is pharmaceutical companies making money; 343 (35.1%) were undecided or did not know and 446 (45.6%) disagreed. Only 53 participants (5.4%) agreed that the main purpose of clinical trials is doctors making money; 197 (20.1%) were undecided or did not know and 680 (69.5%) disagreed.

Most participants (780 [79.8%]) strongly believed in the necessity of informed consent before participating in clinical trials, 139 (14.2%) were undecided, and 28 (2.9%) did not see it as necessary. Relatives providing informed consent was deemed acceptable by 298 participants (30.5%), while 438 (44.8%) disagreed and 209 (21.4%) were unsure. Participants were also asked whether their medical records could be used for scientific purposes without consent: 303 (31.0%) agreed, 430 (44.0%) disagreed, and 210 (21.5%) were unsure.

## Discussion

This study sheds light on a critical challenge in oncology research: the willingness of patients with cancer and their relatives to participate in clinical trials, which is essential for advancing cancer treatment worldwide. Despite the significant role of clinical trials in improving patient outcomes and enabling access to novel therapies, participation rates remain suboptimal globally.^19^ Our findings, which reveal both a willingness to engage and persistent concerns among Turkish patients and families, mirror challenges observed internationally and underscore the universal need for targeted education and transparent communication to bridge gaps in clinical trial participation.

One of the key components for the successful conduct of a clinical trial is the ability to enroll a sufficient number of patients at an adequate pace. A crucial requirement for this is the willingness of patients to participate in the clinical trial. This study offers a thorough analysis of awareness of, perceptions of, and willingness to participate in clinical trials among a demographically diverse cohort of patients with cancer and their relatives in Turkey. The willingness to participate in clinical trials observed in our study (43.8%) is notably higher than that reported in an early study from Turkey.^20^ The study by Taban et al^20^ from 2011 found that only 33.7% of respondents were willing to participate a clinical trial, with significant hesitancy driven by limited knowledge and misconceptions about clinical trials. This difference may be attributed to the broader demographic coverage in our study, which included both patients and their relatives, as well as the higher socioeconomic status and education levels of our respondents. It should also be noted that the aforementioned study^20^ was not conducted specifically in patients with cancer. Still, willingness to participate in clinical trial is notably lower than that observed in other countries. In contrast to our findings, a Canadian survey^22^ reported a 68% willingness to participate in clinical trials, significantly higher than the 43.8% willingness observed in our Turkish cohort. This difference could stem from a higher public awareness of clinical trials in Canada, likely supported by long-standing government and institutional initiatives to promote clinical research.^21^ Additionally, Canada’s universal health care system might influence patient trust and willingness to participate. The Center for Information and Study on Clinical Research Participation survey^14^ from 68 countries reported an even higher willingness of 74.5% globally. In Saudi Arabia, 61% of patients with cancer and their families who were aware of clinical trials expressed willingness to participate,^23^ which is closer to the rates observed in our study. Similar to our results, a study conducted in Lebanon^17^ found that 45% of participants were aware of the concept of clinical trials and 41% expressed a willingness to participate in a phase 1 clinical trial. This finding highlights that in countries with developing health care systems, similar concerns—such as treatment adverse effects and the risk of receiving a placebo—are prevalent. Addressing these universal concerns through educational efforts could enhance participation across diverse regions.

Our findings align with international studies indicating that lack of understanding about clinical trials is a barrier to participation worldwide. A universal approach involving patient education, particularly in less developed health care systems, could help demystify clinical trials and address fears surrounding trial participation.

While our study found no significant gender differences in willingness to participate, global literature often highlights a lower participation rate among women, especially in rural areas. Understanding local barriers to recruitment, such as limited transportation options or family responsibilities, could help tailor interventions to increase inclusivity in clinical trials. The lower willingness to participate in clinical trials observed in less economically developed regions of Turkey might be explained by financial concerns or limited access to trusted health care information. In countries with similar economic challenges, ensuring that patients understand the potential cost-free benefits of trials and addressing any fears about out-of-pocket expenses could be key to improving enrollment.^24,25^

Our data indicate a substantial lack of knowledge about clinical trials among participants, with nearly 60% reporting little to no knowledge. This knowledge gap is a critical barrier to participation, as evidenced by the significant association between well-informed understanding of clinical trials and willingness to participate. Additionally, nearly half of the respondents were uncertain about the safety of clinical trials, underscoring the importance of transparent communication about the risks and safeguards involved. Efforts to enhance public knowledge about clinical trials, particularly regarding their safety and regulatory oversight, could potentially increase participation rates.

Our study also found that participants who had previous experience with clinical trials or had a friend or relative who participated were significantly more willing to engage in new trials. This suggests that positive experiences with clinical trials can help mitigate some of the prevailing negative perceptions, an area that could be leveraged in future recruitment efforts.

The strong motivations identified among participants, such as the belief that clinical trials could improve their illness (61.8%) and the opportunity to access new treatments (53.4%), highlight the potential key points for increasing trial enrollment. Emphasizing these benefits in educational campaigns and patient discussions could be crucial in enhancing participation rates.

The significant concerns about adverse effects (56.7%), the possibility of receiving a placebo (20.1%), and the breach of medical information privacy (11.3%) underline the critical need for transparent communication regarding the risks and safeguards associated with clinical trials. These concerns are consistent with findings from other regions, indicating that safety and privacy are universal barriers that must be addressed to increase participation.^14^ Strategies could include more detailed explanations of the trial process, the role of placebos, and how participant data are protected.

The observed impact of oncologist involvement on patient willingness to participate (71.5% would join if their oncologist was the trial investigator) suggests a trust-based approach that could be applied globally. In many countries, especially those with less established trial frameworks, emphasizing the role of trusted health care professionals as study investigators may enhance participation and alleviate patient concerns.^26^

Future research should explore the cultural and psychological factors that affect clinical trial perceptions and participation across diverse populations. Longitudinal studies are needed to understand how attitudes toward clinical trials evolve over time, especially in response to educational interventions. Expanding research to include different geographic regions and health care settings would provide a more comprehensive view of global barriers and motivators for clinical trial participation. Additionally, developing tailored strategies that address specific concerns, such as safety, trust, and informed consent, could foster a more positive perception of clinical trials and increase patient involvement.

Oncologists play a key role in building patient trust and influencing clinical trial participation. Clear communication that addresses trial safety and benefits can improve patient understanding and willingness to participate. Integrating trial discussions into routine care and training clinicians to discuss trials effectively could enhance enrollment, advancing cancer research with more diverse patient representation.

### Limitations and Strengths

This study has several limitations that should be considered. First, the cross-sectional design restricts our ability to infer causal relationships between identified factors and willingness to participate in clinical trials. Second, data were self-reported, which may introduce recall and social desirability biases, potentially affecting the accuracy of participants’ responses. Third, the sample was drawn from specific regions and health care settings in Turkey, which may limit the generalizability of these findings to other populations or countries with different health care infrastructures. Furthermore, while our study captures key factors informing willingness to participate, it may not account for all potential influences, including unmeasured cultural factors that could affect perceptions of clinical trials.

Despite these limitations, this study offers several notable strengths. It provides a comprehensive assessment of both patients with cancer and their relatives, allowing for a broader perspective on the facilitators and barriers to clinical trial participation. The large sample size and representation of participants from diverse socioeconomic and geographic backgrounds enhance the robustness and relevance of our findings within Turkey. Additionally, our study applies validated methodologies and adheres to STROBE guidelines, ensuring methodological rigor and transparency in reporting. By highlighting both motivations and barriers, our findings provide valuable insights that can inform strategies to increase clinical trial participation, not only in Turkey but also in other countries facing similar challenges.

## Conclusions

This study found significant gaps in knowledge and persistent concerns about clinical trials among patients with cancer and their relatives in Turkey, affecting their willingness to participate. Nearly half of the participants expressed a readiness to engage in clinical trials, yet barriers, such as perceived risks, fear of exploitation, and limited understanding, remain prominent. Our findings underscore the importance of patient-centered education and transparent communication about the safety and benefits of clinical trials. Addressing these factors could enhance trial participation rates, leading to more inclusive and representative cancer research that ultimately improves patient outcomes.

## Notes

Supplement.
Data Sharing Statement
