# Supplementary material for: Understanding of Clinical Trials Among Patients With Cancer and Their Relatives
Source: JAMA Netw Open. 2025 Jan 28;8(1):e2457020. doi: 10.1001/jamanetworkopen.2024.57020 (PMC11775742; doi:10.1001/jamanetworkopen.2024.57020)
Supplement: Supplement. — Data Sharing Statement [file jamanetwopen-e2457020-s001.pdf]

Data Sharing Statement

Kubilay Tolunay. Understanding of Clinical Trials Among Patients With Cancer and Their Relatives. *JAMA Netw Open*. Published January 28, 2025.
doi:10.1001/jamanetworkopen.2024.57020

Data
Data available: No

Additional Information
Explanation for why data not available: The data presented in this study are available on request from the corresponding author. The data are not publicly available due to ethical restrictions.
